# Effect of Human Amniotic Membrane with Aligned Electrospun Nanofiber Transplantation on Tendon Regeneration in Rats

**DOI:** 10.3390/ijms27020650

**Published:** 2026-01-08

**Authors:** Mohamed Nasheed, Mohd Yazid Bajuri, Jia Xian Law, Nor Amirrah Ibrahim

**Affiliations:** 1 Department of Orthopaedics and Traumatology, Faculty of Medicine, Universiti Kebangsaan Malaysia, Cheras 56000, Kuala Lumpur, Malaysia; nasheed_09@hotmail.com; 2 Department of Tissue Engineering and Regenerative Medicine, Faculty of Medicine, Universiti Kebangsaan Malaysia, Cheras 56000, Kuala Lumpur, Malaysia

**Keywords:** human amniotic membrane (HAM), electrospun nanofibers, composite scaffold, tendon healing, gait analysis, histological evaluation, biomaterials

## Abstract

Tendon injuries, whether resulting from trauma, repetitive strain, or degenerative conditions, present a considerable clinical challenge. The natural healing process, which involves inflammatory, proliferative, and remodeling phases, is often inefficient and leads to excessive scar tissue formation, ultimately compromising the mechanical properties of the tendon compared to its native state. This highlights the critical need for innovative approaches to enhance tendon repair and regeneration. Leveraging the regenerative properties of human amniotic membrane (HAM) and electrospun PCL/gelatin nanofibers, this study aims to develop and assess a novel composite scaffold in a rodent model to facilitate improved tendon healing. This prospective experimental study involved 12 male Sprague Dawley rats (250–300 g), randomly assigned to three groups: Group A (No Treatment/No HAM), Group B (HAM-treated), and Group C (HAM with electrospun nanofibers, HAM-NF). A surgically induced tendon injury was created in the left hind limb, while the right limb served as a control. Following surgery, HAM and HAM-NF (0.5 cm^2^) were applied to the respective treatment groups, and tendon healing was assessed after six weeks. Gait analysis, including stride length and toe-out angle, was conducted both pre-operatively and six weeks post-operatively. Macroscopic and microscopic evaluations were performed on harvested tendons to assess regeneration, comparing treated groups to the controls. Gait analysis demonstrated that the HAM-NF group showed a significant increase in stride length from 11.70 ± 1.50 cm to 12.79 ± 1.71 cm (*p* < 0.05), with only a modest change in toe-out angle (14.58 ± 2.96° to 16.27 ± 2.20°). In contrast, the No Treatment group exhibited reduced stride length (10.27 ± 2.17 cm to 8.40 ± 1.67 cm) and a marked increase in toe-out angle (16.33 ± 4.51° to 26.47 ± 5.81°, *p* < 0.05), while the HAM-only group showed mild changes in both parameters. Macroscopic evaluation showed a significant difference in tendon healing. HAM-NF group had the highest score that indicates more rapid tissue regeneration. Histological analysis after 6 weeks showed that tendons treated with HAM-NF achieved a mean histological score of 5.54 ± 4.14, closely resembling the uninjured tendon (6.67 ± 1.63), indicating substantial regenerative potential. The combination of human amniotic membrane (HAM) and electrospun nanofibers presents significant potential as an effective strategy for tendon regeneration. The HAM/NF group exhibited consistent improvements in gait parameters and histological outcomes, closely mirroring those of uninjured tendons. These preliminary results indicate that this biomaterial-based approach can enhance both functional recovery and structural integrity, providing a promising pathway for advanced tendon repair therapies.

## 1. Introduction

Tendon injuries, caused by trauma, repetitive stress, or degenerative conditions, are a significant clinical challenge. These injuries disrupt the structural and mechanical integrity of the tissue, leading to pain, functional impairment, and decline in quality of life [1,2]. The healing process of tendons, comprising inflammatory, proliferative, and remodeling phases, is often inefficient, resulting in scar tissue formation with inferior mechanical properties compared to native tendon tissue [3]. Consequently, the need for innovative strategies to enhance tendon repair and regeneration has become increasingly evident.

Traditional approaches to tendon repair include immobilization, physiotherapy, and surgical interventions such as sutures and grafts. However, these methods are associated with limitations, including a high risk of re-injury, suboptimal functional recovery, and insufficient restoration of mechanical properties [4]. Tissue engineering has emerged as a promising alternative, providing scaffolds that can mimic the extracellular matrix (ECM), support cell attachment, and promote tissue regeneration [5].

Electrospinning is a versatile technique that uses electrostatic forces to produce fine fibers from polymer solutions. It facilitates the production of individual fibers with diameters ranging from 10 nm to 10 µm, spanning micrometer to nanometer scales. The process entails the application of a high-voltage electric field to a polymer solution or melt, overcoming surface tension to create a fine jet of polymer. This jet solidifies or evaporates during its flight, forming a non-woven fabric-like structure composed of nanoscale fibers [6,7]. The resulting scaffolds exhibit high porosity, a large surface area, and excellent structural controllability, making them ideal for various tissue engineering applications, particularly tendon regeneration [8].

A major advantage of electrospinning lies in its ability to produce aligned nanofibers that resemble the native ECM of tendons [9]. These nanofiber membranes, with their high surface-to-volume ratio and interconnected porosity, enhance the attachment and proliferation of tenocytes, the primary cell type in tendons. Additionally, electrospun scaffolds can replicate the mechanical properties of tendons, including their non-linear stress–strain behavior, which is crucial for functional recovery [10]. By tailoring the composition and alignment of the fibers, the mechanical strength, biodegradability, and bioactivity of the scaffold can be optimized to meet the specific requirements of tendon regeneration [11].

Among the materials used in electrospinning, gelatin and polycaprolactone (PCL) are particularly notable for their complementary properties. Gelatin, a derivative of collagen obtained through hydrolysis, mimics the ECM structure of human tissues and offers excellent biocompatibility and biodegradability [12]. It is widely available and adaptable under different temperature and pH conditions, thus establishing it as a versatile material for tissue engineering applications [13]. However, gelatin’s limited mechanical strength and hydrophilicity restrict its standalone use in tendon regeneration [13].

PCL, on the other hand, is a semi-crystalline synthetic polyester with good processability, biodegradability, and mechanical resistance [14]. Its low melting temperature and relatively low cost make it a popular choice for scaffold fabrication [15]. However, PCL is hydrophobic and lacks bioactive molecules, which limits its cellular attachment and proliferation capabilities [16]. By blending PCL with gelatin, the strengths of both materials can be combined. Co-electrospinning these polymers enhances the biocompatibility of PCL while improving the mechanical properties of gelatin, resulting in composite scaffolds with superior mechanical and histological characteristics for tendon regeneration [16]. The use of PCL/GT nanofibers, with their adjustable mechanical properties, enables the creation of a scaffold that can accommodate the dynamic loads exerted on tendon tissue, thereby promoting optimal regeneration and improving integration with the host tissue [17,18].

The human amniotic membrane (HAM) is a biologically derived scaffold known for its rich ECM composition, low immunogenicity, and secretion of bioactive factors [19]. HAM’s biocompatibility and bioactivity make it an appropriate scaffold for tissue engineering, particularly in tendon regeneration. However, its limited mechanical strength necessitates reinforcement with other materials. Combining HAM with aligned electrospun PCL/gelatin nanofibers presents a synergistic approach that leverages their complementary properties. The electrospun nanofibers can enhance the mechanical strength of HAM, while HAM’s bioactivity can improve the cellular attachment and proliferation of the composite scaffold [4]. However, the thin layer of electrospun PCL/gelatin nanofibers deposited on top of the HAM in this study did not contribute significant mechanical strength and primarily served to guide cell alignment and enhance the tenogenic differentiation potential of the cells. In the future, strategies such as preparing thicker electrospun nanofibers (NF) scaffolds on the amniotic membrane or employing crosslinking techniques are needed to enhance the mechanical strength of the HAM/NF scaffold. The benefits of HAM/NF scaffolds for tendon healing have been reported in previous studies [20,21]. The studies reported the use of a multilayer amniotic-PCL nanofibrous membrane with and without celecoxib to prevent post-surgical tendon adhesion.

This novel combination aims to create a versatile scaffold that mimics the structural and biological properties of native tendon tissue. The aligned nanofibers provide the anisotropic architecture necessary for tendon function, while HAM’s bioactive molecules support cell signaling and tissue repair. Such a scaffold has the capacity to overcome the challenges of existing materials and provide a more effective solution for tendon regeneration.

Animal models are essential for translational research in tendon regeneration, allowing the evaluation of scaffolds and therapeutic strategies under controlled conditions. Large animals such as rabbits, dogs, horses, and goats have been used to study tendon repair due to their anatomical similarities to humans [22]. These models enable the replication of standard surgical techniques and the assessment of mechanical properties. However, the high incidence of postoperative tendon re-tear in large animals complicates the interpretation of regenerative outcomes [23].

Rodent models, particularly rats, offer several advantages for tendon repair studies. Their anatomical resemblance to human tendons, coupled with the absence of postoperative re-tear, makes them ideal for mechanistic and therapeutic investigations [2,24]. Additionally, the availability of transgenic and lineage-tracing models in rodents facilitates the study of cellular and molecular mechanisms underlying tendon healing. These models serve as valuable platforms for screening regenerative therapies during the initial discovery phases.

Given the promising properties of HAM, electrospun nanofibers, and rodent models, this study aims to fabricate and evaluate a novel composite scaffold for tendon regeneration. The scaffold will combine HAM with aligned electrospun PCL/gelatin nanofibers to create a biomimetic structure that addresses the mechanical and biological requirements of tendon repair. By using a rat model, the present study assessed the scaffold’s efficacy in promoting tendon regeneration at macroscopic and microscopic levels. This approach demonstrates promise in advancing the field of tendon tissue engineering and provides improved therapeutic options for patients with tendon injuries.

Therefore, this study aims to evaluate the potential of using human amniotic membrane (HAM) combined with aligned polycaprolactone/gelatin (PCL/GT) nanofibers to promote tendon regeneration, an approach that has not been reported previously.

Specifically, the objectives of this study are:

1. To compare the gait performance of rats before and after HAM–NF (electrospun nanofiber) transplantation.
2. To evaluate the regenerated tendon tissue both macroscopically and microscopically following implantation.

## 2. Results

### 2.1. Gait Analysis Results

**Figure 1:**
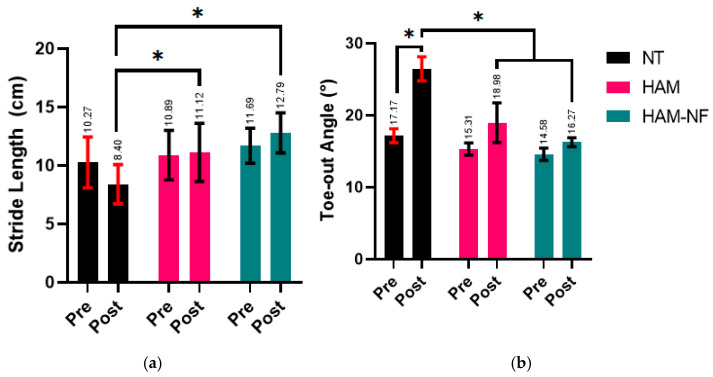
(**a**) Comparison of average stride length between three groups pre- and post- surgery: non-treatment (NT), human amniotic membrane (HAM), HAM with electrospun nanofiber (HAM-NF), and (**b**) Toe-out angle between the three groups pre- and post- surgery. Error bar in SD. * *p* < 0.05 (2-Way ANOVA).

The gait analysis, including stride length and toe-out angle, was evaluated pre- and post-treatment across all groups, with no significant differences observed at baseline (Table 1). Significant post-treatment improvements were observed in specific parameters for the treatment groups (Figure 1). The HAM/NF group showed a significant increase in stride length from 11.70 ± 1.50 cm pre-treatment to 12.79 ± 1.71 cm post-treatment (*p* < 0.05). The toe-out angle also increased slightly from 14.58 ± 2.96° to 16.27 ± 2.20° post-treatment, indicating improved gait parameters. In the HAM-only treated group, the stride length showed a slight reduction from 10.89 ± 2.11 cm to 11.12 ± 2.49 cm while the toe-out angle increased from 15.30 ± 2.94° to 18.98 ± 9.59°, suggesting a mixed response with potential biomechanical adaptations post-treatment. The No HAM group demonstrated the most pronounced changes in the toe-out angle, which significantly increased from 16.33 ± 4.51° pre-treatment to 26.47 ± 5.81° post-treatment (*p* < 0.05), with the stride length decreased from 10.27 ± 2.17 cm to 8.40 ± 1.67 cm, indicating potential compensatory mechanisms or limited recovery in this group.

Overall, the HAM/NF group exhibited consistent improvements in gait parameters, particularly in stride length, highlighting its potential in promoting tendon repair and restoring functional mobility. The significant differences between pre- and post-treatment outcomes further underscore the efficacy of the HAM/NF treatment in enhancing gait recovery.

### 2.2. Gross Morphological Scoring

**Figure 2:**
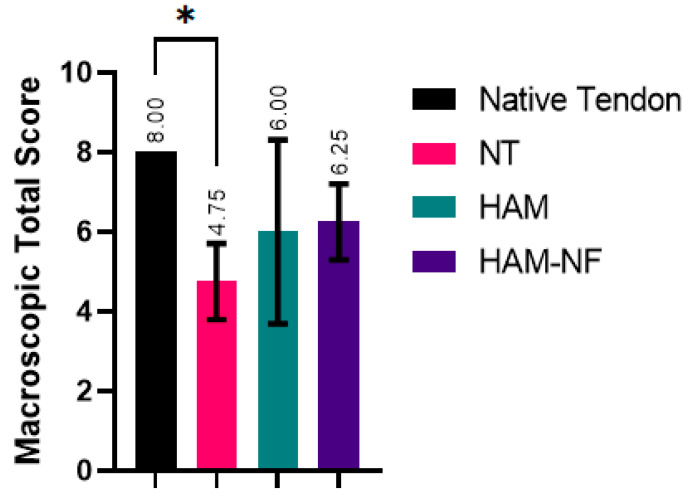
The macroscopic total score based on gross examination after tendons were harvested six weeks post-surgery to assess visual and structural characteristics such as color, surface structure, shape and size of tendon between the positive control (native tendon), non-treated group (NT), and treatment groups (HAM and HAM-NF). Error bar in SD. * *p* < 0.05 (One-way ANOVA).

**Figure 3:**
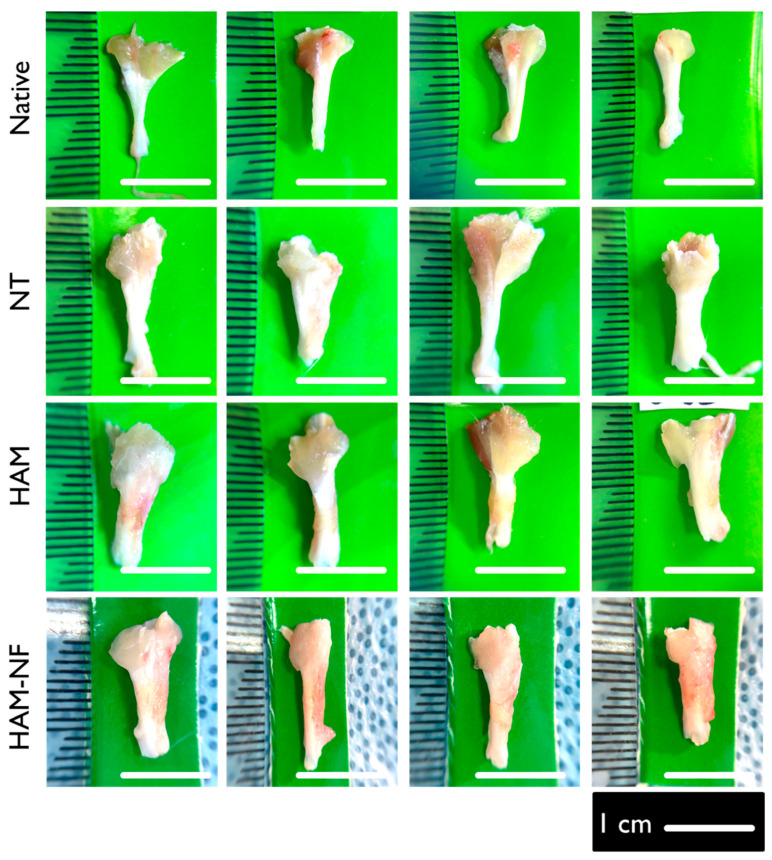
Gross appearance of tendon tissue harvested six weeks post-surgery from positive control (native), non-treated (NT), and treatment groups (HAM and HAM-NF) for macroscopic assessment.

The macroscopic evaluation of tendon healing showed distinct differences among the groups (Table 2). Further visual comparisons are shown in Figure 2. The native tendon exhibited the highest macroscopic score (8.00 ± 0.00), serving as the reference for optimal tendon structure. The HAM-NF group demonstrated the best healing among the experimental groups (6.25 ± 0.95), followed closely by the HAM group (6.00 ± 2.30). In contrast, the No Treatment (NT) group had the lowest score (4.75 ± 0.95, *p* = 0.005), indicating the poorest healing outcome. Visual inspection revealed that tendons in the HAM-NF and HAM groups exhibited better structural integrity compared to the NT group, which showed more irregularities (Figure 3). The findings suggest that HAM-NF transplantation promotes improved tendon healing, while the absence of a biological scaffold results in significantly impaired regeneration.

### 2.3. Association Between Treatment Groups with Uninjured Tendon

**Figure 4:**
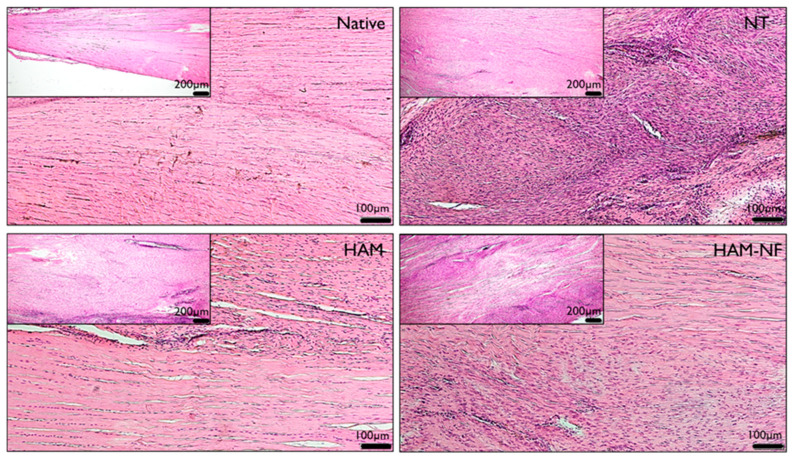
Hematoxylin and Eosin (H&E) stain of tendon tissue of all groups, including positive control native tendon, non-treated (NT), and treatment groups (HAM and HAM-NF). The images were captured in 4X (top, left) at a scale of 200 µm and 10X magnification at a scale of 100 µm.

The histological scoring was conducted following the method outlined by Stoll et al [25]. to assess the quality of the regenerating tissue and the response to HAM/NF transplantation. The histological scoring of tendons after 6 weeks of treatment revealed significant differences between the treatment groups and the uninjured tendon (Figure 4). As shown in Table 3, the uninjured tendon, serving as the control group, exhibited the highest histological score (6.67 ± 1.63), indicating superior structural integrity and normal tendon histology. In contrast, tendons treated with Human Amniotic Membrane combined with electrospun nanofibers (HAM/NF) showed a mean histological score of 5.54 ± 4.14. While this score was lower than that of the uninjured tendon, there was no statistically significant difference between rats treated with HAM/NF and the uninjured tendon, suggesting that HAM/NF contributes significantly to tendon healing. This improvement may be attributed to the synergistic effects of the human amniotic membrane and the electrospun nanofibers, which provide a conducive microenvironment for cellular proliferation and extracellular matrix remodeling, enhancing the regenerative potential [26]. Tendons treated with HAM alone had a mean histological score of 4.55 ± 0.52, which was significantly lower than that of the uninjured tendon (*p* < 0.05). This result indicates that HAM alone offers partial improvement in histological characteristics but is less effective than HAM/NF in restoring tendon integrity. The group without HAM treatment (No HAM) had the lowest histological score (4.40 ± 0.54), significantly different from the uninjured tendon (*p* < 0.05). This finding highlights the limited natural healing capacity of tendons without any supportive treatment. HAM/NF’s superior performance in promoting tendon healing may be explained by its ability to mimic the native extracellular matrix and provide structural and biochemical support. The amniotic membrane contains growth factors, cytokines, and extracellular matrix components that facilitate anti-inflammatory effects, angiogenesis, and fibroblast recruitment [27]. Meanwhile, the electrospun nanofibers enhance mechanical strength and provide a scaffold for cellular attachment and proliferation, further promoting tissue repair [28]. Together, these components synergistically improve tendon healing, resulting in histological scores closer to the uninjured tendon. These findings underscore the clinical potential of HAM/NF as an effective treatment strategy for tendon repair.

## 3. Discussion

Tendon injuries pose a significant clinical challenge due to their limited capacity for self-repair, often resulting in fibrosis and compromised mechanical properties. Various biological scaffolds have been explored to enhance tendon regeneration, with human amniotic membrane (HAM) showing promising potential due to its bioactive components. The integration of aligned electrospun nanofibers (HAM/NF) further enhances the regenerative process by mimicking the native extracellular matrix (ECM), providing structural support and guiding cellular organization. This preliminary study aimed to evaluate the potential of HAM/NF transplantation in promoting tendon regeneration, with the hypothesis that HAM/NF would enhance tendon healing by improving functional recovery, macroscopic structural integrity, and histological characteristics compared to HAM alone or no treatment. The findings strongly support this hypothesis. In the gait analysis, the HAM/NF group exhibited a significant increase in stride length post-treatment (*p* < 0.05), suggesting improved functional recovery, whereas the No HAM group demonstrated gait abnormalities, including a significant increase in toe-out angle (*p* < 0.05), indicating compensatory biomechanical changes due to impaired tendon healing. Macroscopic evaluation further reinforced these results, as the HAM/NF-treated tendons had the highest macroscopic healing score among the treatment groups and were closest to the native tendon, while the No HAM group exhibited significantly poorer healing (*p* = 0.005). Histological analysis also revealed that the HAM/NF group had a histological score closest to the uninjured tendon, suggesting improved ECM organization, reduced degenerative changes, and better vascularization. In contrast, the No HAM group had significantly lower histological scores (*p* < 0.05), confirming inadequate tendon regeneration in the absence of treatment.

The mechanistic basis behind the beneficial effects of HAM/NF in tendon repair can be more clearly understood by considering the biological complexity of the human amniotic membrane and the structural contribution of aligned nanofibers. The human amniotic membrane contains epithelial and mesenchymal stem cells embedded within a collagen-rich extracellular matrix, together with growth factors and cytokines that collectively exert anti-inflammatory, angiogenic, immunomodulatory, and anti-fibrotic effects, thereby supporting accelerated wound healing and reducing scar formation [28]. When aligned electrospun fibers are incorporated onto HAM, as demonstrated by Hasmad et al. [26], the resulting composite scaffold exhibits higher tensile strength and provides uniaxial topographical cues that guide cell alignment along the direction of fiber orientation. In the context of tendon healing, this synergy between HAM’s bioactive milieu and the nanofibers’ anisotropic structure offers a mechanistic explanation for the improved ECM organization, reduced degenerative changes, and superior functional gait outcomes observed in the HAM/NF group.

The regenerative advantages of the HAM/NF scaffold likely arise from the coordinated delivery of biochemical and biomechanical cues that replicate key aspects of native tendon repair microenvironments. Aligned electrospun nanofibers mimic the anisotropic architecture of native tendon ECM, providing a topographical guide that promotes tenocyte or progenitor cell elongation and alignment, and directs collagen type I deposition along the fiber axis rather than random scar-like organization [29]. When seeded with appropriate cells (e.g., stem cells), such aligned fibers upregulate tendon-specific markers (e.g., scleraxis, tenomodulin, COL1) and encourage a tenogenic phenotype, as opposed to non-aligned or random scaffolds. Beyond structural guidance, aligned nanofiber scaffolds also improve the mechanical environment: by bearing load in a directionally appropriate manner, they reduce strain irregularities and provide mechanical stability during remodeling, which supports organized ECM synthesis under physiologic loads. Moreover, when such fibrous scaffolds are combined with bioactive membranes (such as HAM) or supplemented with growth factors, they can deliver sustained biochemical stimuli (e.g., growth factors, immunomodulatory signals) that promote cell migration, proliferation, controlled inflammation, and angiogenesis [9].

Finally, the scaffold’s porous, nanofibrous network supports nutrient and oxygen diffusion and facilitates vascular ingrowth—a prerequisite for sustaining the metabolic demands of active ECM synthesis and remodeling. Taken together, these biochemical, topographical, and mechanical signals cooperate to trigger mechanotransductive pathways, steer tenogenic differentiation, and support collagen organization and vascularized tissue formation—thereby promoting regeneration over fibrosis and underpinning the superior structural and functional healing observed with HAM/NF treatment.

The improvement in stride length in the HAM/NF group suggests more effective restoration of tendon function. This response is likely influenced by the structural guidance provided by the aligned nanofibers, which support organized tissue formation and help reduce maladaptive remodeling. These combined effects contribute to smoother gait mechanics and better overall functional recovery [30,31]. Scar tissue can limit mobility and negatively impact gait performance, but aligned nanofibers have been shown to facilitate more effective tissue regeneration due to their capacity to mimic the natural extracellular matrix experienced in tendon structures [32,33]. The mechanical properties of aligned nanofibers, improving tensile strength and elasticity, provide a superior biomechanical environment conducive to functional recovery post-injury. Moreover, the presence of growth factors and bioactive elements inherent in human amniotic membrane may have played an essential role in enhancing cellular activities critical for tendon repair, which further supports improved biomechanical functions observed in gait analysis [34,35]. This can lead to a functional restoration of the tendon and a concurrent improvement in gait characteristics. The advancements in stride length within the HAM/NF cohort are consistent with findings reported in other studies examining the benefits of biomimetic scaffolding for tendon repair. For instance, aligned nanofibers have been shown to provide mechanical support that not only aids in cellular adhesion and proliferation but also reduces the formation of fibrotic tissue, allowing for more functional healing [18,31]. This alignment of nanofibers directs cellular orientation and ultimately enhances the biomechanical properties of the regenerated tendons, which is crucial for restoring movement patterns akin to those observed prior to injury.

The smaller change in toe-out angle suggests that, despite improvements in tendon recovery, its effect on rotational mechanics during gait was more modest. This pattern indicates that certain aspects of limb kinematics may require longer healing time or may be less responsive to early structural repair [36,37]. Variation in toe-out angle among the groups likely reflects the complex interplay between tendon recovery and gait adaptation. The marked increase seen in untreated animals points toward greater reliance on compensatory movement patterns, indicating insufficient tendon restoration and altered load distribution during ambulation [37,38]. The altered load distribution associated with increased toe-out angles can affect how forces are transmitted throughout lower limb joints [39,40].

Macroscopic assessment demonstrated clearer structural restoration in the HAM/NF group, reflecting the scaffold’s ability to support more organized tissue repair. Although none of the treated tendons fully matched native morphology, HAM/NF showed the closest resemblance, underscoring its potential to enhance gross healing quality. This pattern is consistent with evidence that bioactive scaffolds can improve tissue organization and overall repair outcomes in tendon regeneration [41]. Specifically, the ability of HAM to provide a rich environment for cellular proliferation and the structural advantages of NF, to support tensile strength and mechanical properties, are significant facets contributing to observed healing enhancements [42]. Although the HAM/NF group did not fully restore the tendon to optimal native conditions, its score indicates substantial improvement and better healing than the untreated groups. The ability of tendon injuries to recover fully often hinges on the preservation and restoration of biomechanical properties, which are critical in determining functional outcomes. Studies have indicated that scaffolds, particularly those leveraging the biochemical and mechanical properties present in natural tissues, can markedly influence the structure and integrity of regenerated tendon tissues [20]. Notably, the healing scores provided by our macroscopic assessment resonate with findings in similar studies where the use of electrospun scaffolds led to tissue regeneration. Research has shown that carefully designed nanofibrous scaffolds possess anisotropic properties that mimic the extracellular matrix (ECM) characteristics, which are crucial for facilitating cellular interactions and promoting organized tissue repair. Such scaffolding offers advantages by ensuring appropriate mechanical support during the healing phase, enabling enhanced load-bearing capabilities and biomechanical functioning post-repair. A fundamental mechanism contributing to the observed improvements in the HAM/NF group stems from the properties of the human amniotic membrane itself, which is well documented for containing growth factors that stimulate cellular activities pivotal in the healing process [43]. These growth factors, such as epidermal growth factor (EGF) and vascular endothelial growth factor (VEGF), have been shown to enhance cell proliferation and migration, thereby fostering the repair process of tendons and modulating inflammation effectively [44]. This biocompatibility and inherent ability to secrete important growth factors render HAM an invaluable component in regenerative medicine. The electrospun nanofibers also play a critical role in enhancing mechanical properties while encouraging tenocyte alignment, thereby facilitating better organization of the regenerated tissue [11]. The aligned structure of nanofibers can help improve tensile strength and elasticity, providing a supportive landscape for tendon regeneration. This organization is crucial since tendons require specific mechanical properties to withstand dynamic loads encountered during movement; therefore, scaffolds that can deliver both biochemical and biomechanical support are of paramount importance. Moreover, electrospun scaffolds have been noted for their capacity to enhance the healing environment by creating favorable microenvironments around the injured site. For instance, the interconnected porosity of electrospun nanofibers allows for adequate nutrient and waste exchange, supporting cellular metabolism and ultimately improving tissue integration [41].

Histological assessment showed that HAM/NF produced tissue architecture more closely resembling healthy tendon compared to other treated or untreated groups. This pattern suggests that the combined scaffold better supports organized matrix remodeling and cellular activity essential for tendon restoration. While HAM alone offered partial benefit, the composite approach demonstrated clearer regenerative potential [41], emphasizing the inadequacy of natural healing mechanisms without any supportive intervention. Thus, the results signify that utilizing HAM/NF treatment can markedly advance tendon healing as opposed to relying solely on intrinsic repair processes. A critical aspect of the histological evaluation involves the examination of ECM organization and cell–matrix interactions within the tendon healing context. The results indicated that ECM organization was better preserved in tendons treated with HAM/NF when compared with other groups. Properly structured collagen fiber deposition is essential for the mechanical properties and functionality of tendons. The findings suggest that the electrospun nanofibers facilitated enhanced alignment and organization of collagen fibers, which is fundamental in maintaining the structural integrity of the tendon. Enhanced cell adhesion due to the nanofibrous structures further supports effective matrix remodeling and cellular responses, which are crucial for tendon regeneration. These findings align with earlier studies exploring composite scaffolds combining human amniotic membrane and aligned electrospun fibers. Hasmad et al. [26] reported that electrospun PLGA fibers deposited on decellularized HAM formed a mechanically competent scaffold with enhanced tensile strength in the hydrated state and uniaxially aligned fibers that directed skeletal muscle cell orientation. Such anisotropic fiber architecture closely resembles the parallel collagen bundle arrangement essential for load-bearing tissues, supporting proper mechanotransduction and functional recovery. Similarly, the biological activity of HAM—stemming from its growth factors, cytokines, and stem-cell-derived components—creates a regenerative microenvironment that modulates inflammation and limits fibrosis [28]. In our tendon model, the combination of structural guidance from the aligned nanofibers and the pro-healing bioactivity of HAM provides a coherent mechanistic rationale for the superior histological organization and functional gait restoration observed in the HAM/NF group. Electrospun nanofibers have been documented to contribute positively to the cellular environment by providing a surface that mimics the natural architecture of the tendon ECM, thus promoting tenocyte attachment and growth. This effect helps to create an organized environment conducive to ECM production and restructuring, which is supported by various studies showing that engineered scaffolds can improve healing outcomes.

The histological analysis revealed that the HAM/NF group exhibited reduced degenerative changes relative to the NT group, which portrayed poorer tissue structure. The presence of degenerative changes frequently indicates suboptimal healing and structural disruption [45]. In particular, the HAM/NF treatment helped in minimizing tissue degeneration, suggesting that it provided an effective regenerative environment that supports cellular health and function.

In terms of inflammation, the results showed that while the HAM/NF group had higher inflammation markers compared to uninjured tendons, this observation does not imply detrimental effects. On the contrary, heightened inflammation in this context can indicate an active remodeling process essential for healing rather than chronic inflammation [41]. Inflammatory responses are critical during the early healing phases, as they facilitate appropriate cellular mobilization and establish an environment conducive to tissue regeneration. Conversely, the untreated group displayed diminished inflammation, which could signify inadequate healing characterized by fibrotic repair mechanisms rather than robust tendon regeneration. Understanding the interplay between inflammation and fibrosis in tendon healing is essential to developing new therapies that encourage proper tendon recovery without excessive scar tissue formation [46].

Vascularization plays a vital role in tendon healing, as it is essential for providing nutrients and oxygen to the healing tissue. The histological evaluation indicated that HAM/NF treatment resulted in higher vascularization compared to uninjured tendons. The presence of angiogenic growth factors in the human amniotic membrane likely stimulates vascular development, further enhancing the healing capacity of the tendons. Several studies have established that improved vascularity correlates with better healing outcomes in tendon injuries, reinforcing the significance of angiogenesis for effective tissue repair. The integration of biomaterial scaffolds, such as the one utilized in the HAM/NF treatment, has been documented to significantly enhance vascularization and lead to improved healing responses in various tendon models. The contribution of vascularization to tendon healing cannot be overstated, as increased blood supply promotes the overall reparative processes, including ECM turnover and cellular activity critical for functional recovery [47].

The impact of HAM/NF on tendon healing can be better understood through its inherent properties and biochemical composition. The human amniotic membrane contains a wealth of growth factors, including VEGF, EGF, and transforming growth factor-beta (TGF-β), which collectively promote fibroblast mobilization, ECM remodeling, and angiogenesis. This remarkable bioactivity establishes an environment rich in regenerative signals necessary for effective wound healing. Moreover, the low immunogenicity associated with HAM supports optimal healing by minimizing adverse inflammatory responses that could impair the regenerative process. This ability to facilitate enhanced healing while preventing excessive inflammation is crucial for effective tendon repair [48]. The incorporation of electrospun nanofibers within the HAM scaffolding provides mechanical support while simultaneously enhancing cell alignment and adhesion, leading to organized ECM deposition. The presence of electrospun nanofibers mimics the native tendon ECM, thereby promoting physiological cellular interactions essential for optimal tendon healing [46].

When comparing these findings to existing literature, it is evident that the composite use of HAM and electrospun nanofibers offers distinct advantages over traditional biomaterials and techniques employed in tendon regeneration. The mechanisms behind effective tendon regeneration involve coordinated cellular activities and favorable mechanical properties provided by bioengineered scaffolds [49].

In prior studies involving other biomaterials, findings indicated that while single-component treatments yielded some positive effects, they often failed to restore the full structural and functional capacity of the tendon, potentially leading to re-injury or chronic dysfunction. The combined application of HAM and nanofibers, as demonstrated in this study, represents a mechanistically sound approach to enhancing tendon healing and optimizing recovery.

One key limitation of this study is the short follow-up period, which primarily focused on early tendon healing. Tendon regeneration is a prolonged process, and long-term studies are necessary to determine whether the observed improvements persist and translate into functional strength comparable to native tendon tissue. Future studies should include extended follow-up periods to evaluate the durability of the repaired tendons. Additionally, the small sample size and use of a rat model pose constraints in terms of clinical applicability. While rodent models are valuable for initial investigations, they do not fully replicate the biomechanical and physiological complexities of human tendons. Future research should incorporate larger animal models, such as rabbits, sheep, or pigs, which better mimic human tendon mechanics, before progressing to clinical trials. We acknowledge that the absence of blinding constitutes a limitation of the study and may introduce potential bias. Nonetheless, all evaluations were performed according to predefined, standardized criteria to minimize subjectivity. Another limitation is the lack of biomechanical strength evaluation in this study. While functional improvements were assessed through gait analysis, a detailed examination of tendon tensile strength and elasticity was not performed. Understanding the mechanical resilience of regenerated tendons is crucial for clinical application, as tendons must withstand physiological loading. Future studies should include tensile strength testing and failure load assessments to confirm the robustness of HAM/NF-repaired tendons. On top of that, the lack of mechanical data of HAM and HAM/NF scaffolds used in this study is also a limitation that warrants further investigations. Nonetheless, the tensile strength of HAM has been reported to range from 2 to 10 MPa, which is at the lower end of native tendon tissue (5–100 MPa) [7,11,41]. The thin layer of NF deposited on top of the HAM did not provide significant mechanical strength. In the future, the analysis of tendon-specific markers such as collagen type I/III, tenomodulin, or scleraxis would further strengthen the confirmation of tendon regeneration rather than general tissue repair. Furthermore, while HAM/NF already provides a supportive environment for tendon repair, combining it with additional bioactive factors, such as platelet-rich plasma (PRP), growth factors (e.g., TGF-β, VEGF), or stem cells, may further accelerate healing and enhance regenerative capacity. Studies should explore multi-modal treatment approaches to optimize outcomes. To enhance clinical translation, future research should focus on long-term functional and biomechanical studies to evaluate the effectiveness of HAM/NF beyond the early healing phase. Additionally, transitioning to large animal models will be essential for validating its therapeutic potential. Incorporating mechanical testing, including tensile strength assessments, load-bearing capacity evaluations, and stress–strain analysis, will ensure that the regenerated tendon meets functional criteria suitable for human applications. By addressing these limitations and optimizing scaffold-based strategies, HAM/NF transplantation promises to be a viable and innovative approach for tendon repair, potentially improving patient outcomes and reducing the burden of chronic tendon injuries.

## 4. Materials and Methods

### 4.1. Research Approval

Approval to conduct research in Hospital Canselor Tuanku Muhriz (HCTM) were approved by the Secretariat Research and Innovation, Faculty of Medicine, Universiti Kebangsaan Malaysia (UKM), reference number UKM.FPR.SPI800-2/28 with grant code FF-2023-322. This study was also approved by the University Kebangsaan Malaysia Animal Ethics Committee (UKMAEC) (UKM.PPI.AEC.800-4/3/1) with approval number; ORTH/FP/2023/MOHD YAZID/26-JULY/1354-AUG.-2023-AUG-2023 on the 16th of August 2023.

### 4.2. Study Design

This study was a prospective experimental study designed to evaluate the effects of human amniotic membrane (HAM) and HAM with electrospun nanofibers (HAM-NF) on tendon regeneration in a Sprague Dawley rat model. The study involved 12 male Sprague Dawley rats, each weighing 250–300 g, which underwent a surgically induced Achilles tendon injury. The rats were randomly divided into three groups: Group A (Control—No HAM), Group B (HAM-treated), and Group C (HAM/NF-treated). Each rat had a tendon injury in the left hind limb, while the right hind limb served as an internal control. The tendons were evaluated six weeks postoperatively to assess the effects of the treatment.

### 4.3. Fabrication of Composite EF-HAM Scaffolds

Decellularized, air-dried human amniotic membrane (amnion) (5 cm × 5 cm) purchased from the USM Tissue Bank (Malaysia) was attached to the collector prior to electospinning. The fabrication of composite PCL/GT nanofibers was performed as previously described by Lim et al., 2021 [41]. Nanofibers were prepared using the electrospinning method using a combined PCL (Sigma-Aldrich, Saint Louis, MO, USA) and GT (Nitta Gelatin, Japan) mixture. Briefly, PCL was dissolved in 2,2,2-trifluoroethanol (TFE; Sigma) to prepare 6% (*w*/*v*) solution. The solution was magnetically stirred for 24 h until the polymers dissolved completely. GT was dissolved using TFE to prepare a 10% (*w*/*v*) solution. A mixture of the solution was then prepared at a 70:30 ratio. The solution mixture was electrospun at an applied voltage of 5 kV, a flow rate of 0.05 mL/h, 45 min of spinning duration, and 20 cm needle-to-collector distance onto a 1000 rpm rotating collector that yielded aligned nanofibers without beadings. The resulting HAM with PCL/GT nanofibers was airdried under a biosafety cabinet to allow for complete solvent evaporation, UV-irradiated, and soaked with 1% antibiotic-antimycotic solution, followed by incubation in complete culture medium prior to any experimental use.

### 4.4. Surgical Procedure

**Figure 5:**
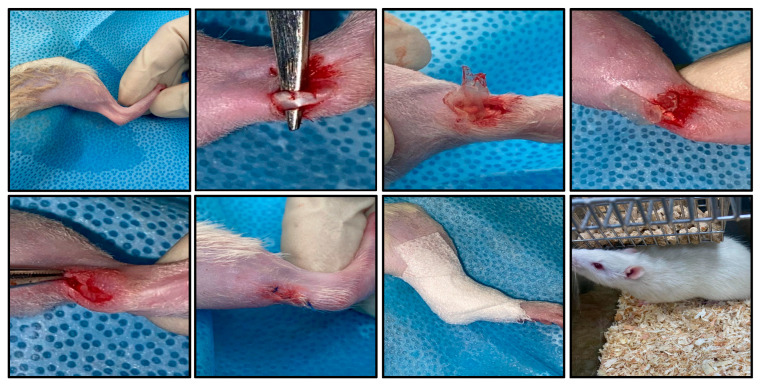
Key steps of the surgical procedure.

Rats were anesthetized using Ketamine/Xylazine (Mavlab, Australia/Troy Laboratories, Australia, and the distal portion of the right hind limbs was shaved and disinfected with chlorhexidine gluconate solution (Rinscap, Malaysia). The surgical steps are depicted in Figure 5. A longitudinal skin incision, centered over the Achilles tendon, approximately 5 mm proximal to the calcaneum, was made to expose the tendon. A transverse hemisection of the tendon was performed about 2 mm proximal to the Achilles insertion using a No. 15 scalpel blade, while care was taken to avoid injury to the flexor hallucis longus tendon, which lies medial to the Achilles tendon. Treatment was then applied according to group allocation. Group A received no treatment, Group B had HAM (0.5 cm^2^) wrapped around the injured tendon, and Group C had HAM-NF (0.5 cm^2^) wrapped around the injured tendon. Following treatment application, the wound was closed using proline 6-0 sutures, and the rats were placed in a warm environment until they regained consciousness. Postoperative care included oral administration of paracetamol (200 mg/kg) via drinking water every 24 h for three days to manage pain and discomfort.

### 4.5. Assessment and Evaluation

#### 4.5.1. Gait Analysis

**Figure 6:**
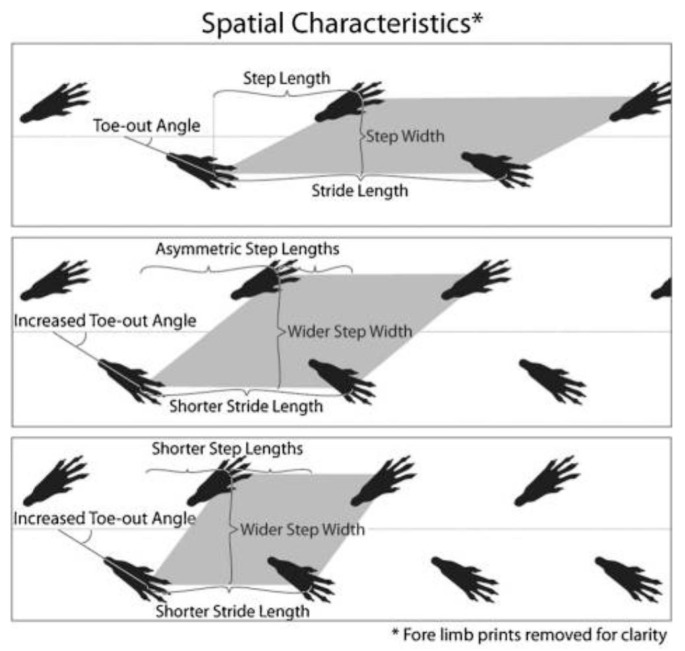
Gait analysis methods.

Gait analysis was conducted pre-operatively and six weeks post-operatively to assess functional recovery following tendon injury and treatment. The hind limb paws of the rats were painted with Chinese ink and allowed to walk on a 40 cm paper strip to obtain paw prints. These paper strips were then used to record gait parameters, including stride length and toe-out angle (Figure 6). The average for stride length and toe-out angle was measured for each rat; the pre-op and the post-op measurements were compared between the groups to determine the effectiveness of HAM and HAM-NF in restoring normal gait patterns.

#### 4.5.2. Macroscopic Evaluation

At six weeks post-surgery, the tendons were harvested and examined macroscopically to assess visual and structural characteristics. The evaluation focused on the color, surface structure, shape, and size of the repaired tendon. These macroscopic findings provided an initial assessment of tendon healing across the different treatment groups. the macroscopic assessment was adapted from Lohan et al., 2011 (Table 4) [50].

#### 4.5.3. Microscopic Evaluation

Histological analysis was performed using the Modified O’Driscoll Histological score (Table 5) to evaluate tendon regeneration at the cellular level. Parameters assessed included the total number of cells, the number of tenocytes, the parallel orientation of cells, vascularization, and collagen fiber arrangement. Specifically, the amount of fibers with large diameters and parallel orientation was examined to determine the structural integrity of the regenerated tendon.

### 4.6. Statistical Analysis

All collected data from gait analysis, macroscopic evaluation, and histological scoring (*n* = 4) were analyzed using one-way ANOVA with Tukey’s post hoc test to compare differences between groups. The significance level was set at *p* < 0.05.

## 5. Conclusions

This study demonstrates the potential of human amniotic membrane with aligned electrospun nanofibers (HAM/NF) transplantation in promoting tendon regeneration. The findings indicate that HAM/NF enhances functional recovery, improves macroscopic tendon healing, and supports histological integrity, making it a promising candidate for tendon repair strategies. Gait analysis revealed that HAM/NF transplantation significantly improved stride length, suggesting better biomechanical recovery compared to untreated tendons. Macroscopic evaluation further confirmed superior healing in the HAM/NF-treated group, which exhibited more rapid tendon regeneration. Histological assessment showed that HAM/NF treatment resulted in a more organized extracellular matrix, reduced degenerative changes, and improved vascularization, indicating its potential to facilitate tendon remodeling. Despite these promising results, several limitations must be addressed before clinical application. The short follow-up period, small sample size, and lack of biomechanical strength evaluation highlight the need for further research. Future studies should focus on long-term tendon remodeling, biomechanical assessments, and large animal models to confirm the durability and mechanical resilience of the regenerated tendon tissue. Additionally, exploring combinatorial approaches with bioactive molecules or growth factors may further enhance tendon regeneration. Overall, this study provides compelling evidence that HAM/NF transplantation supports tendon healing by providing a bioactive and biomechanically favorable environment. With further optimization and validation in preclinical models, HAM/NF could serve as an innovative and clinically viable approach for tendon repair, potentially improving patient outcomes and reducing the burden of tendon-related injuries.

## Figures and Tables

**Table 1:** Stride length and toe-out angle before and after treatment.

Variables	Group (Mean ± SD, cm)			
	Stride Length		Toe-Out Angle (°)	
	Pre-	Post-	Pre-	Post-
HAM/NF	11.70 ± 1.50	12.79 ± 1.71 *	14.58 ± 2.96	16.27 ± 2.20
Ham Only	10.89 ± 2.11	11.12 ± 2.49	15.30 ± 2.94	18.98 ± 9.59
No Ham	10.27 ± 2.17	8.40 ± 1.67	16.33 ± 4.51	26.47 ± 5.81 *

* *p* < 0.05 when compared to pre-surgery.

**Table 2:** Macroscopic Scoring.

Groups	Macroscopical Score (Mean ± SD)	*p*-Value (Compared to Native Tendon)
Native Tendon	8.00 ± 0.00	
HAM/NF	6.25 ± 0.95	0.089
Ham Only	6.00 ± 2.30	0.056
No Ham	4.75 ± 0.95	0.005 *

* indicates statistical significance of *p* < 0.05.

**Table 3:** Histological Scoring of Tendon.

Variables	Group (Mean ± SD, cm)			
	Uninjured Tendon	HAM/NF	Ham Only	No Ham
Total Histological Score	6.67 ± 1.63	5.54 ± 4.14	4.55 ± 0.52 *	4.40 ± 0.54 *
ECM organization	1.50 ± 0.54	1.00 ± 0.00	1.00 ± 0.00	1.00 ± 0.00
Cell/Matrix Ratio	1.00 ± 0.00	1.00 ± 0.00	1.00 ± 0.00	0.80 ± 0.45
Cell Distribution	0.33 ± 0.54	0.18 ± 0.40	0.45 ± 0.52	0.40 ± 0.54
Organization of Repair Tissue	1.00 ± 0.00	1.00 ± 0.44	1.00 ± 0.00	1.00 ± 0.00
Degenerative Changes	2.33 ± 0.82	1.36 ± 0.50	1.00 ± 0.00	1.00 ± 0.00
Vascularization	0.00 ± 0.00	0.36 ± 0.50	0.09 ± 0.00	0.4 ± 0.00
Inflammation	0.50 ± 0.54	0.63 ± 0.67	0.09 ± 0.30	0.00 ± 0.00

* *p* < 0.05 when compared to uninjured tendon.

**Table 4:** Macroscopic Scoring based on [50].

Macroscopical Scoring	Points
Color	
White, glossy	2
Rose, rough	1
Other color	0
Surface structure	
Smooth, tight, intact	2
Rough	1
Surface with cleft and holes	0
Shape	

**Table 5:** Histological Scoring System.

Histological Criteria	Description	Points
Extracellular Matrix (ECM) Organization	Collagen fibers are wavy, compact, and arranged in parallel.	2
	Collagen fibers are partially compact and partially loose.	1
	Collagen fibers are loosely composed and accompanied by granulation tissue.	0
Cell/Matrix Ratio	The ratio of cells to matrix is physiological (normal).	2
	There is a locally increased cell density.	1
	Cell density is abnormally high.	0
Cell Distribution	Cells are distributed homogeneously and physiologically.	1
	Cells show a heterogeneous distribution with clustering.	0
Organization of Repair Tissue	The repair tissue is homogeneously organized.	2
	The repair tissue is locally heterogeneous.	1
	The composition of the tissue has completely changed.	0
Degenerative Changes/Tissue Metaplasia	No degenerative changes are present.	3
	Moderate edema formation is observed.	2
	Intense edema with gap formation is present.	1
	There is evidence of cartilage or bone assembly.	0
Vascularization	The tissue is hypo-vascularized (reduced blood vessel formation).	1
	The tissue is hyper-vascularized (increased blood vessel formation).	0
Inflammation	No inflammatory cells are detected.	1
	Inflammatory cells, such as neutrophils, macrophages, or giant cells, are present.	0
Maximum Total Score	Represents optimal tissue repair conditions.	12

## Data Availability

The original contributions presented in this study are included in the article. Further inquiries can be directed to the corresponding author.

## Abbreviations

The following abbreviations are used in this manuscript:

HAM	Human Amniotic Membrane
HAM-NF	HAM with electrospun nanofibers
ECM	Extracellular Matrix
PCL	Polycaprolactone
PCL/GT	Polycaprolactone/gelatine
AMSC	Amniotic mesenchymal stromal cells
AEC	Amniotic epithelial cells
EGF	epidermal growth factor
bFGF	basic fibroblast growth factor
VEGF	vascular endothelial growth factor
TGF	transforming growth factor
PDGF	platelet-derived growth factor
MSC	mesenchymal stromal cells
TIMP	tissue inhibitors of metalloproteinase
TEM	Transmission electron microscopy
SEM	scanning electron microscopy
